# Dupilumab for Adult and Adolescent Patients With Primary Eosinophilic Colitis

**DOI:** 10.14309/ctg.0000000000000908

**Published:** 2025-08-26

**Authors:** Twan Sia, Leeon Bacchus, Aparna Kumar, Lina Fikri, Rachel Solecki, Ramin Herath, Yeelin Bacchus, Audrey Apollon, Margaret Werd, Aseelah Ashraf, Elaine Wu, Dan Bahar, John Leung

**Affiliations:** 1 Stanford University School of Medicine, Stanford, California, USA;; 2 Boston Specialists, Boston, Massachusetts, USA;; 3 University of Massachusetts Chan Medical School, Worcester, Massachusetts, USA.

**Keywords:** eosinophilic colitis, eosinophilic gastrointestinal disorder, dupilumab, upadacitinib

## Abstract

**INTRODUCTION:**

Eosinophilic colitis (EoC) currently has limited options to induce histologic and clinical remission. Dupilumab is a human monoclonal antibody against the interleukin-4 receptor ɑ subunit effective in eosinophilic esophagitis (EoE) and other non-EoE eosinophilic gastrointestinal disorders.

**METHODS:**

We conducted a retrospective chart review to assess the histoclinical response of patients with EoC treated with dupilumab.

**RESULTS:**

Of 4 included patients, all 4 improved histologically, with 3 patients attaining histologic remission. All patients' symptom scores significantly improved, and 1 patient was asymptomatic on dupilumab.

**DISCUSSION:**

This retrospective case series provides preliminary evidence that dupilumab may be an effective treatment option for EoC.

## INTRODUCTION

Primary eosinophilic colitis (EoC) is a disorder characterized by eosinophilic inflammation in the colon with lower gastrointestinal tract symptoms such as diarrhea or abdominal pain (1). While EoC in infants can be managed by temporary elimination diet, adolescent and adult patients with EoC require more aggressive medical management (1). Current treatment options are limited to elimination diet, corticosteroids, and immunomodulatory agents (1).

Dupilumab, a human monoclonal antibody against the interleukin-4 receptor alpha subunit, is effective for treating eosinophilic esophagitis (EoE) (2) and other non-EoE eosinophilic gastrointestinal disorders (3). However, to date, dupilumab has never been described in the treatment of EoC. In this retrospective cohort study, we evaluate the effects of dupilumab in patients with EoC.

## METHODS

We conducted a retrospective chart review at a single multidisciplinary clinic, examining patients with EoC who were treated with dupilumab. Although there are no consensus criteria for histologic remission of EoC, the Consortium of Eosinophilic Gastrointestinal Disease Researchers (CEGiR) suggests active EoC should be defined as ≥65 eosinophils/high-power field (eos/hpf) in the sigmoid colon, ≥80 eos/hpf in the descending colon, and ≥100 eos/hpf in the ascending colon (4). Primary endpoints for this study were clinical, histologic, and endoscopic changes pre-dupilumab versus on dupilumab.

Protected health information, dates, and mentions of treatment were removed using the available National Library of Medicine's NLM-Scrubber tool as previously described (5,6). Three blinded investigators then scored symptoms pre-dupilumab and on dupilumab to form a symptom score, with a minimum symptom score of 0, and a maximum of 18 (most severe symptoms).To ensure interrater reliability, an intraclass correlation coefficient was calculated using a two-way mixed-effects model with absolute agreement. Intraclass correlation coefficient was 0.89, which was acceptable reliability based on *a priori* criterion (ICC ≥0.70). Additional details are available in Supplemental Methods and Supplementary Table 1 (http://links.lww.com/CTG/B369).

## RESULTS

From 15 patients identified by *ICD-10* code, 3 did not have histologic confirmation of EoC, 7 did not try dupilumab, and 1 had a secondary cause of colonic eosinophilia (ulcerative colitis). Thus, 4 patients were included in our study. All patients were female. Three were adults, and 1 was an adolescent at the time of diagnosis (Table 1).

**Table 1.** Clinical presentations of patients at baseline and after treatment with dupilumab

Patient	Age at diagnosis (yr)	Race/Ethnicity Sex	Treatment [indication]	Failed treatments	Comorbid EGIDs	Atopic comorbidities	Pretreatment symptoms	Average symptom score (pretreatment →on dupilumab)
1	15.5	Non-Hispanic White, F	Dupilumab 300 mg weekly [eosinophilic esophagitis]	2FED	EoE, EoD	None	Abdominal pain, nausea and vomiting, weight loss	8.67 → 5.67
2	21	Non-Hispanic white, F	Dupilumab 300 mg biweekly, loading dose 600 mg [asthma]	2FED	None	Asthma, atopic dermatitis	Diarrhea, abdominal pain	4.00 → 2.67
3	73.5	Non-Hispanic white, F	Dupilumab 300 mg biweekly, loading dose 600 mg [atopic dermatitis]	Swallowed budesonide 9 mg daily	None	Atopic dermatitis, allergic rhinitis	Diarrhea, abdominal pain, fatigue	4.67 → 2.33
4	33.5	Non-Hispanic white, F	Dupilumab 300 mg weekly [eosinophilic esophagitis]	Swallowed fluticasone 880 mcg twice daily; swallowed mometasone 1.6 mg twice daily	EoE	Asthma, allergic rhinitis, food allergies (fish, flaxseed, sesame seeds, kiwi)	Diarrhea, nausea, and vomiting	4.33 → 0

2FED, 2 food elimination diet (milk and wheat elimination diet); EGID, eosinophilic gastrointestinal disease; EoD, eosinophilic duodenitis; EoE, eosinophilic esophagitis; Eos/hpf, eosinophils per high-power field.

At baseline, all patients were symptomatic: diarrhea (n = 3), abdominal pain (n = 3), nausea/vomiting (n = 2), and weight loss (n = 1) (Table 1). On colonoscopy, all patients had visually normal mucosa. However, biopsies revealed significant colonic eosinophilic infiltration, raising suspicion for EoC (Table 2). Further workup for secondary causes of EoC were negative for all patients. Two patients were treated with milk and wheat food elimination diet (2 food elimination diet) and the other 2 patients with various swallowed steroids, none with symptom improvement (Table 1). Patient 4 had multiple colonoscopies on fluticasone (prescribed for EoE) with no change in eosinophilic infiltration.

**Table 2.** Changes in eosinophil count per high-power field by location in the colon

Patient	Treatment	Rectum	Sigmoid colon	Descending colon	Transverse colon	Ascending colon	Cecum	Terminal ileum
Patient 1	Pretreatment					45 eos/hpf	100 eos/hpf^a^	
	On dupilumab	17 eos/hpf	22 eos/hpf	19 eos/hpf	25 eos/hpf	28 eos/hpf	22 eos/hpf	45 eos/hpf
Patient 2	Pretreatment	48 eos/hpf	60 eos/hpf	111 eos/hpf^a^	125 eos/hpf^a^	100 eos/hpf^a^	95 eos/hpf^a^	
	On dupilumab		40 eos/hpf	50 eos/hpf	48 eos/hpf	52 eos/hpf	39 eos/hpf	26 eos/hpf
Patient 3	Pretreatment			100 eos/hpf^a^		100 eos/hpf^a^		
	On dupilumab		60 eos/hpf	60 eos/hpf	70 eos/hpf	100 eos/hpf^a^		
Patient 4	Pretreatment			120 eos/hpf^a^		100 eos/hpf^a^		40 eos/hpf
	On dupilumab			11 eos/hpf		52 eos/hpf		9 eos/hpf

Eos/hpf, eosinophils per high power-field.

a Indicates histologically active based on Consortium of Eosinophilic Gastrointestinal Disease Researchers (CEGiR) suggested criteria.

Then, patients were switched to treatment with dupilumab a median of 24.29 weeks after diagnosis (min-max 4.29–230.71, Q1–Q3 4.71–90.46), 2 for active, co-occurring EoE (300 mg weekly), and atopic comorbidities (300 mg biweekly, loading dose 600 mg) (Table 1). Patients were on dupilumab for a median of 33.93 weeks (min-max 13.14–50.14, Q1–Q3 25.04–41.68) before repeat colonoscopic and clinical evaluation.

All patients' colonic eosinophilia improved on repeat colonoscopy on dupilumab. All patients except for Patient 3 achieved histologic remission (Table 2).

Median symptom score at baseline was 4.5 (min–max 4.0–8.67, Q1-Q3 4.25–5.67). After treatment with dupilumab, all patients' symptom scores significantly improved to a median of 2.5 (min-max 0–5.67, Q1–Q3 1.75–3.42; two-tailed paired T-test *P* = 0.0221). One patient (Patient 4) was completely asymptomatic on dupilumab (Table 1; Supplementary Table 2, http://links.lww.com/CTG/B369). Although we were unable to identify when patients began experiencing clinical improvement after starting dupilumab, a longer treatment duration was nonsignificantly associated with improved symptoms scores (Pearson's *r* = −0.93, *R*^2^ = 0.86, β = −0.07, *P* = 0.6) and improved peak eosinophil count (Pearson's *r* = −0.81, *R*^2^ = 0.66, β = −1.54, *P* = 0.19). Although neither association reached statistical significance, this is likely because of our small sample size.

Two patients later switched from dupilumab to upadacitinib, a selective Janus kinase 1 inhibitor. Patient 1 had great histologic improvement and some clinical benefit with dupilumab; however, she had persistent breakthrough symptoms (symptom score 5.67 on dupilumab). In addition, she had co-occurring EoE nonresponsive to dupilumab after 89.14 weeks of treatment (Table 3). She was switched to upadacitinib, which has been shown to have efficacy in treating EoC (7) and EoG in case reports (8). Patient 3 switched to upadacitinib because of dupilumab-induced conjunctivitis despite clinical improvement of EoC. After 11.0 weeks, she had a symptom score of 1.0 (compared with 2.33 on dupilumab). None of the other patients had any adverse effects attributable to dupilumab.

**Table 3.** Changes in eosinophil count per high-power field by location in the esophagus for patients with co-occurring eosinophilic esophagitis

Patient	Treatment	Proximal esophagus	Middle esophagus	Distal esophagus
Patient 1	Pre-treatment	55 eos/hpf^a^	100 eos/hpf^a^	50 eos/hpf^a^
	On-dupilumab	3 eos/hpf	5 eos/hpf	100 eos/hpf^a^
Patient 4	Pre-treatment	50 eos/hpf^a^	13 eos/hpf	10 eos/hpf
	On-dupilumab	0 eos/hpf	0 eos/hpf	0 eos/hpf

Eos/hpf, eosinophils per high power-field.

a Indicates histologically active eosinophilic esophagitis.

## DISCUSSION

In conclusion, we report the use of dupilumab for EoC in 3 adult and 1 adolescent patient, showing improvement in histologic and clinical endpoints. Current treatments for EoC are limited. In addition, dupilumab is the first-line treatment for EoE, which is a comorbidity in 10.9% of patients with EoC (9). A further 41.8% of patients with EoC have other atopic comorbidities that may be treated with dupilumab (9). Thus, dupilumab may be considered as an ideal treatment over antitumor necrosis factor agents such as infliximab or adalimumab in these patients. Our research suggests that both weekly and biweekly administration of dupilumab can be efficacious, but larger studies are required to determine the optimum dosing schedule.

JAK inhibitors have been shown to be effective for EoC in a recent series of 4 patients (6), likely by inhibiting downstream Th1 and Th2 signaling cascades. In comparison, dupilumab is primarily inhibits Th2-mediated inflammation, such as in EoE. Although 1 patient (patient 3) had improved symptoms on upadacitinib compared with dupilumab, it is unclear whether blocking broader inflammatory signaling with JAK inhibitors will always result in greater symptom response compared with dupilumab. Further confirmatory studies with well-designed prospective trials are needed.

## CONFLICTS OF INTEREST

**Guarantor of the aricle:** Dr. John Leung.

**Specific author contributions:** Sia T: Conceptualization, Formal Analysis, Investigation, Methodology, Supervision, Validation, Visualization, Writing – Original Draft Preparation, Writing – Review & Editing, Bacchus L: Conceptualization, Data Curation, Formal Analysis, Investigation, Methodology, Validation, Visualization, Writing – Original Draft Preparation, Writing – Review & Editing, Kumar A: Data Curation, Formal Analysis, Investigation, Validation, Visualization, Writing – Original Draft Preparation, Writing – Review & Editing, Fikri L: Data Curation, Formal Analysis, Investigation, Validation, Visualization, Writing – Original Draft Preparation, Writing – Review & Editing, Solecki R: Data Curation, Formal Analysis, Investigation, Validation, Visualization, Writing – Original Draft Preparation, Writing – Review & Editing, Herath R: Data Curation, Formal Analysis, Investigation, Validation, Visualization, Writing – Original Draft Preparation, Writing – Review & Editing, Bacchus Y: Investigation, Validation, Visualization, Writing – Original Draft Preparation, Writing – Review & Editing, Apollon A: Investigation, Validation, Visualization, Writing – Original Draft Preparation, Writing – Review & Editing, Werd M: Investigation, Validation, Visualization, Writing – Original Draft Preparation, Writing – Review & Editing, Ashraf A: Data Curation, Investigation, Validation, Writing – Review & Editing, Wu E: Data Curation, Investigation, Validation, Writing – Review & Editing, Bahar D: Data Curation, Investigation, Validation, Writing – Review & Editing, Leung J: Conceptualization, Investigation, Methodology, Project Administration, Supervision, Validation, Writing – Original Draft Preparation, Writing – Review & Editing

**Financial support:** None to report.

**Potential competing interests:** J.L. is a paid speaker for AbbVie, Regeneron, Sanofi, and GlaxoSmithKline. He is a consultant for Sanofi, Takeda, Genentech, AstraZeneca, Regeneron, AbbVie, Guidepoint and Bristol Myers Squibb. He receives research funding for various clinical trials from AstraZeneca, Allakos, Bristol Myers Squibb/Celgene, Ellodi Pharmaceuticals, Revolo Biotherapeutics, Sanofi, Takeda, Celldex Therapeutics, Provention Bio, GlaxoSmithKline (GSK), AbbVie, Phathom Pharmaceuticals, Uniquity Bio, Evommune. None of the other authors have relevant conflicts of interest to disclose.

**Data sharing statement:** All relevant deidentified data and study materials are stored in a HIPPA-compliant, password protected, cloud-based storage. Access to these files will be provided upon reasonable request to the corresponding author.

## Supplementary Material

SUPPLEMENTARY MATERIAL
