# Supplementary material for: Dupilumab for Adult and Adolescent Patients With Primary Eosinophilic Colitis
Source: Clin Transl Gastroenterol. 2025 Aug 26;16(10):e00908. doi: 10.14309/ctg.0000000000000908 (PMC12543260; doi:10.14309/ctg.0000000000000908)
Supplement: SUPPLEMENTARY MATERIAL [file ct9-16-e00908-s001.docx]

**METHODS**

The electronic medical record at a single medical center in the northeastern United States, Boston Specialists, was searched between 2017-2025 using the International Classifications of Disease, Tenth Revision (ICD-10) code K52.82 (EoC). This clinic is staffed by board-certified allergist/immunologists and/or gastroenterologists, including those with specialized interest in EGIDs.

Exclusion criteria were (1) patients without histologic or symptomatic evidence of EoC; (2) never started dupilumab; or (3) had other causes for colonic eosinophilia, such as parasitic/helminthic infection, medication effects, or other diseases associated with colonic eosinophilia (e.g. inflammatory bowel disease, rheumatologic disease, or hypereosinophilic syndrome). Though there are no consensus criteria for histologic remission of EoC, the Consortium of Eosinophilic Gastrointestinal Disease Researchers (CEGiR) suggests active EoC should be defined as ≥65 eosinophils/high-power field (eos/hpf) in the sigmoid colon, ≥80 eos/hpf in the descending colon, and ≥100 eos/hpf in the ascending colon.^4^

Symptoms at baseline versus on dupilumab were compared to evaluate if dupilumab improved clinical features of EoC. To minimize rater bias, one investigator collected progress notes at baseline and on-dupilumab. The on-dupilumab timepoint coincided with repeat colonoscopic evaluation. Then, protected health information, dates, and mentions of treatment were removed using the available National Library of Medicine’s NLM-Scrubber tool, as previously described for a study on EoE, though with symptoms that are appropriate for EoC.^5,6^ Next, 3 trained investigators blinded to the study hypothesis scored symptoms (diarrhea, abdominal pain, nausea/vomiting, weight loss, hematochezia, fatigue) on a 4-point scale (0=“Absent”, 1=“Mild”, 2=“Moderate”, 3=“Severe”), which were summed and averaged between the 3 raters. Thus, minimum symptom score was 0, and maximum was 18 (most severe symptoms). The detailed symptom grading criteria by symptom is provided in **Supplementary Table 1.** To ensure interrater reliability, an intraclass correlation constant (ICC) was calculated using a two-way, mixed effects model with absolute agreement. ICC was 0.89, which was acceptable reliability based on *a priori* criterion (ICC≥0.70).

Descriptive statistics were used to analyze clinical characteristics. Primary endpoints were change in mean symptoms score and peak eosinophil counts pre-dupilumab versus on dupilumab. Comparisons of primary endpoints were made using two-way paired t-tests. We used Pearson correlation and linear regression to explore associations between treatment duration with dupilumab and the two primary endpoints.

**Supplementary Table 1.** Symptom grading criteria using a 4-point ordinal scale. BS = Bristol Scale.

| **Symptom** | **Grade 0 (None)** | **Grade 1 (Mild)** | **Grade 2 (Moderate)** | **Grade 3 (Severe)** |
| --- | --- | --- | --- | --- |
| **Diarrhea** | - No diarrhea,  normal bowel movements. | - 1–2 extra bowel movements/day;  - Slightly loose stool (BS 5-6);  - No urgency  - No major impact on daily life. | - 3–5 extra bowel movements/day  - Loose/watery stool (BS 6-7)  - Some urgency  - Mild impact on activities. | - ≥6 extra bowel movements/day or incontinence  - Loose/watery stool (BS 6-7)  - Significant urgency  - Major impact on activities or sleep. |
| **Abdominal Pain** | - No pain. | - Occasional mild (1-3 out of 10) discomfort  - No impact on activities. | - Frequent or moderate (4-6 out of 10) pain  - Limits some activities  - OTC medication used. | - Constant or severe (7-10 out of 10) pain  - Limits most activities  - Requires  prescription meds or emergency care. |
| **Nausea** | - No nausea or vomiting | - Occasional nausea and/or rare vomiting  - Normal eating  - No impact on functioning. | - Frequent nausea and/or occasional vomiting  - Reduced appetite  - May limit activities  - May use anti-nausea meds. | - Persistent nausea and/or frequent vomiting  - Unable to eat or function normally  - Dehydration  - Significant impact on daily life. |
| **Weight Loss** | - No unintentional weight loss. | <5% weight loss over 1–3 months; no functional impact. | 5–10% weight loss; reduced strength or appetite; impacts activity. | >10% weight loss; poor appetite or weakness; requires nutritional support. |
| **Hematochezia** | - No visible blood. | - Small streaks on toilet paper or stool  - Occasional occurrence | - Blood mixed with stool or visible in the bowl  - Frequent occurrence | - Heavy or frequent bleeding  - May require hospitalization |
| **Fatigue** | - No fatigue, normal energy. | - Occasional tiredness  - Rest helps  - No functional impact. | - Fatigue most days - Limits some activities | - Constant, disabling fatigue;  - Unable to do many daily tasks |

**Supplementary Table 2.** Individual and composite symptom scores at baseline and after treatment with dupilumab.

| **Patient** | **Treatment** | **Diarrhea** | **Abdominal Pain** | **Nausea and vomiting** | **Weight loss** | **Hematochezia** | **Fatigue** | **Composite**  **Symptom Score** |
| --- | --- | --- | --- | --- | --- | --- | --- | --- |
| **Patient 1** | **Pre-treatment** | 0 | 3 | 2.67 | 3 | 0 | 0 | 8.67 → 5.67 |
|  | **On-dupilumab** | 0 | 2.67 | 3 | 0 | 0 | 0 |  |
| **Patient 2** | **Pre-treatment** | 1.33 | 2.67 | 0 | 0 | 0 | 0 | 4.00 → 2.67 |
|  | **On-dupilumab** | 0 | 1.33 | 1.33 | 0 | 0 | 0 |  |
| **Patient 3** | **Pre-treatment** | 3 | 1.33 | 0 | 0 | 0 | 0.33 | 4.67 → 2.33 |
|  | **On-dupilumab** | 1.33 | 1 | 0 | 0 | 0 | 0 |  |
| **Patient 4** | **Pre-treatment** | 2.67 | 0 | 1.67 | 0 | 0 | 0 | 4.33 → 0 |
|  | **On-dupilumab** | 0 | 0 | 0 | 0 | 0 | 0 |  |
